# Sleeping posture recognition using fuzzy c-means algorithm

**DOI:** 10.1186/s12938-018-0584-3

**Published:** 2018-11-06

**Authors:** Rong-Shue Hsiao, Tian-Xiang Chen, Mekuanint Agegnehu Bitew, Chun-Hao Kao, Tzu-Yu Li

**Affiliations:** Department of Electronic Engineering, National Taipei University of Technology, No. 1, Sec. 3, Zhongxiao East Road, Taipei, 10608 Taiwan, ROC

**Keywords:** Force-sensing resistor, Infrared array sensor, Sleeping posture recognition, Fuzzy logic

## Abstract

**Background:**

Pressure sensors have been used for sleeping posture detection, which meet privacy requirements. Most of the existing techniques for sleeping posture recognition used force-sensitive resistor (FSR) sensors. However, lower limbs cannot be recognized accurately unless thousands of sensors are deployed on the bedsheet.

**Method:**

We designed a sleeping posture recognition scheme in which FSR sensors were deployed on the upper part of the bedsheet to record the pressure distribution of the upper body. In addition, an infrared array sensor was deployed to collect data for the lower body. Posture recognition was performed using a fuzzy c-means clustering algorithm. Six types of sleeping body posture were recognized from the combination of the upper and lower body postures.

**Results:**

The experimental results showed that the proposed method achieved an accuracy of above 88%. Moreover, the proposed scheme is cost-efficient and easy to deploy.

**Conclusions:**

The proposed sleeping posture recognition system can be used for pressure ulcer prevention and sleep quality assessment. Compared to wearable sensors and cameras, FSR sensors and infrared array sensors are unobstructed and meet privacy requirements. Moreover, the proposed method provides a cost-effective solution for the recognition of sleeping posture.

## Background

Sleeping posture is one of the most important factors that determines sleep quality, reducing sleep disorders and preventing ulcer formation. As treatment of established pressure ulcers is extremely difficult and costly, the ideal solution is prevention. Nursing homes and hospitals use set programs to avoid the development of bed-sores in bedridden patients, such as pressure-shifting on a regular basis and using cushions with pressure-relief components. The sleeping posture of bed-bound patients needs to be regularly changed in order to reduce the risk of developing pressure ulcers [1], and bed sensor systems have been developed to recognize sleeping postures.

Sleeping posture recognition is also an important issue in medical care for ambulatory patients or outpatients, as deterioration or amelioration of certain diseases are related to sleeping posture. For example, researchers have shown that sleeping in the decubitus position increases the risk of developing subacromial impingement syndrome by 3.7× as compared with those who sleep in the supine position [2]. Sleeping in a lateral position can effectively reduce symptoms in patients with mild or moderate sleep apnea [3]. Symptoms of sleep paralysis usually occur in the supine position [4]. Sleeping on the right side has a higher risk than sleeping on the left side of development of transient lower esophageal sphincter relaxation, which is a main factor in nocturnal gastroesophageal reflux [5, 6].

Researchers have also found that sleep quality is related to sleeping position and frequent sleep postural changes. For example, snoring or extensive body movement may result in a shorter sleep duration [7]. Subjects who report a history of poor sleep quality spend more time in the supine position with the head straight [8], and subjects who sleep on their left side have a significantly higher rate of nightmares (40.9%) than those who sleep on their right side (14.6%). Also, recent research in rats indicated that a lateral sleep posture is more effective for waste removal from the brain during sleep [9], while waste in the brain may contribute to some neurological diseases such as Alzheimer’s and Parkinson’s.

For bed-bound and limited-mobility patients, the sensor system employed is usually simple and inexpensive, but may achieve a high posture recognition accuracy when the patient is placed by the caregiver in the central axis of the bed. However, the accuracy drops acutely with deviation of the patient’s body from the central axis [1, 10]. For ambulatory patients or healthy elderly people, the design becomes much more complicated and relatively more expensive, as human beings exhibit a variety of postures during sleep.

In early studies, researchers obtained sleeping posture information by interviewing subjects. In recent years, however, multiple approaches have been developed based on different sensing modalities to determine sleeping postures [11, 12]. Among the various widely-used techniques, pressure-sensing and camera-based visual data are most common. Different camera systems are used to acquire visual data. A common digital camera, described in [13], is used mainly as a fall-detection system, but can also identify a lying posture. In [14], the authors modeled the human body in terms of its constituent body parts; then, for each part, a multi-view Eigen model that combined image views from numerous calibrated cameras was built. Using a deformable triangulation method, a body part segmentation algorithm was presented in [15] based on body postures. However, these methods require high-resolution camera images or a sequence of high-resolution video recordings, and concerns have been raised regarding video monitoring systems in terms of invasion of privacy. In [16–18], the researchers used pressure sensors to determine sleeping posture based on the pressure distribution. In [19], Wai et al. proposed a 56-sensor layout for patients with higher mobility. This scheme consisted of a 7 × 7 round-sensor array for the upper body and a 7 × 1 elongated sensor array for the lower body. The sleeping posture classification accuracy of this system was reported to be 93%.

As most of the pressure distribution is contributed by the hips and chest, limbs are rarely recognized unless thousands of sensors are deployed; in addition, the positions and postures of the feet are more variable than those of the chest and hips. These are the reasons for which the accuracy of FSR-based posture recognition is significantly low. To increase the accuracy and lower the cost, in this work we deployed a new sensing system over the lower body instead of a pressure sensor array. This paper presents a sleeping posture recognition scheme based on force-sensing resistor (FSR) and infrared array sensors. FSR sensors were deployed to obtain the pressure distribution of the upper body, while the lower body position was detected by a single infrared array sensor. In summary, the contribution of this paper is the novel design of an accurate, cost-effective technique for the recognition of sleeping posture.

## Methods

**Fig. 1:**
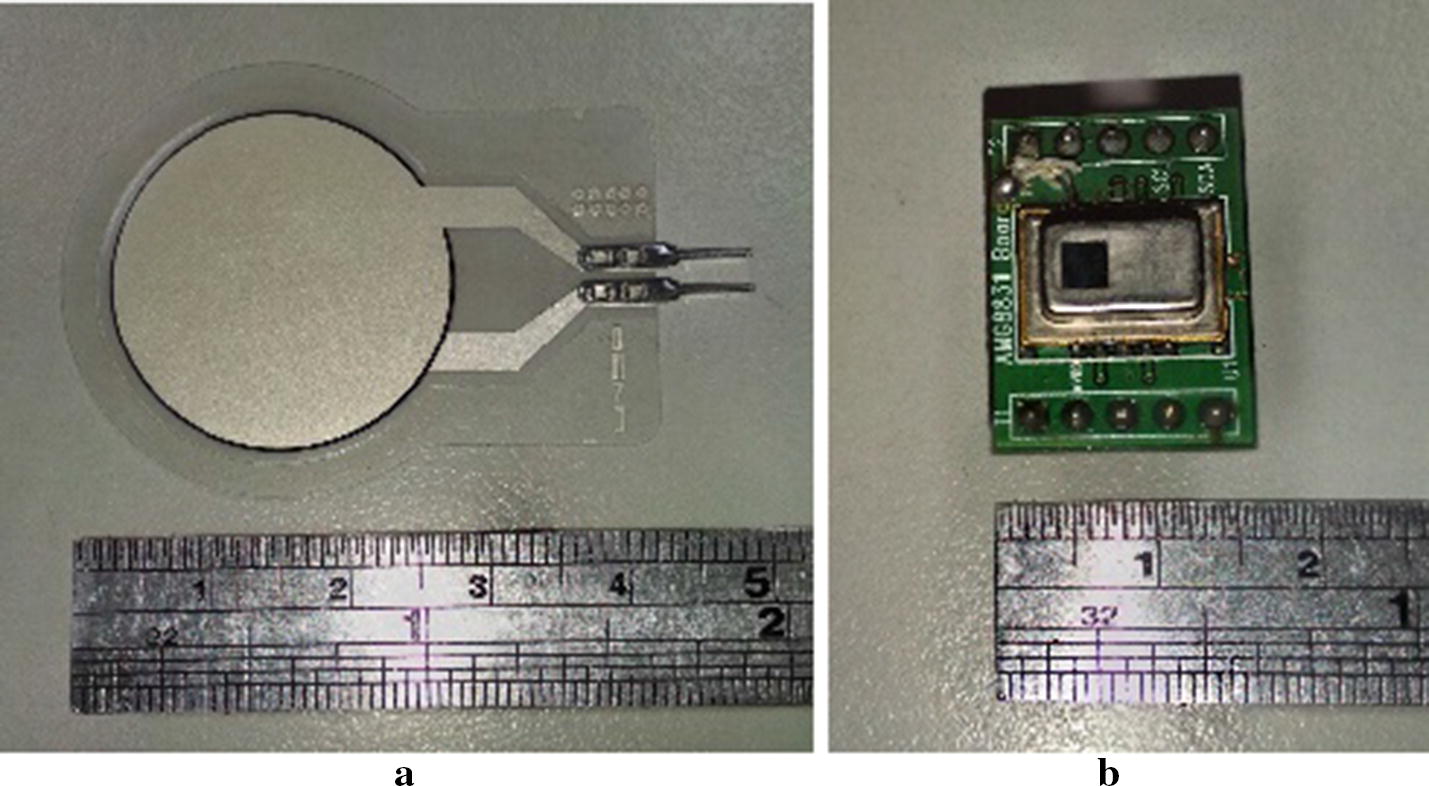
Sensor systems employed in this study: **a** FSR sensors; **b** infrared array sensor

**Fig. 2:**
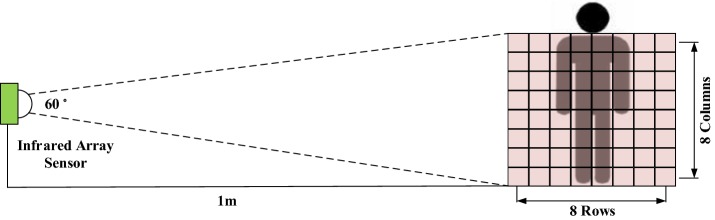
Grid-EYE detection system

The proposed sleeping posture recognition method uses two kinds of sensor: FSR sensors for the upper body and an infrared array sensor (Grid-EYE) for the lower body. FSR sensors are thin-film sensors made of a piezoelectric polymer, and the resistance decreases in proportion to the applied force on the active surface [20]. Each FSR sensor can measure force up to a value of 10 kg/cm^2^. The Grid-EYE system is an infrared thermal imaging array sensor consisting of an 8 × 8 matrix of sensors [21] that detects and renders thermal images of the temperature distribution of a movable or non-movable object. The FSR sensor array used in our experiment is shown in Fig. 1a; the infrared array sensor is shown in Fig. 1b. The sensing coverage of each pixel changed when the distance between the Grid-EYE array and the subject varied, as shown in Fig. 2.

**Fig. 3:**
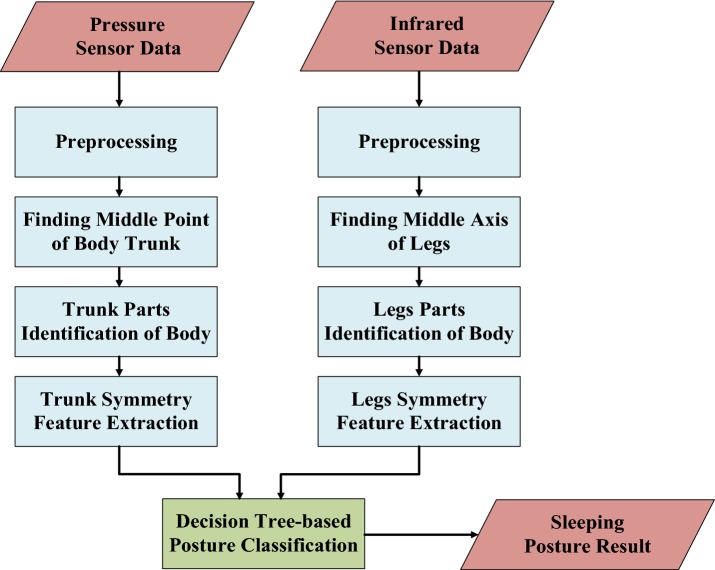
Different levels of data-processing for sleeping posture recognition

**Fig. 4:**
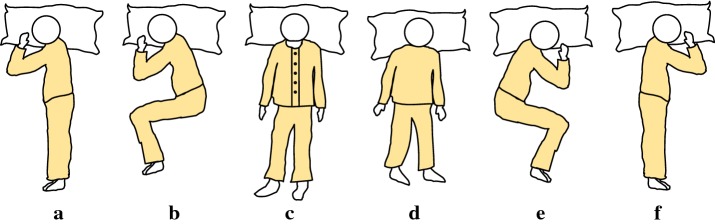
Sleeping posture classification: **a** right log; **b** right fetus; **c** supine; **d** prone; **e** left fetus; **f** left log

Figure 3 shows the different levels of data-processing required for sleeping posture recognition. The sensor data from the pressure and infrared sensor arrays were processed separately to extract the features from each sensor type. The features extracted from both types of sensor were then combined into the final system in order to recognize and distinguish six kinds of sleeping posture, as shown in Fig. 4.

As human beings exhibit a variety of sleeping postures, the postures are usually grouped into several categories based on the body trunk and limb positions. Idzikowski [22] conducted a sleeping position preference survey of around 1000 people in Britain and classified the favorite sleeping postures into six categories. This is the most commonly-used classification of sleeping posture. Liu et al. [18] further classified the fetus (sleeping on one’s side with the body curled up) and the log (sleeping on one’s side with the back and legs straight) positions into left fetus, right fetus, left log, and right log positions, as shown in Fig. 4. This classification method was adopted in our experiments.

### Pre-processing

This section presents the pre-processing method used for both the pressure and infrared sensor data. The purpose of pre-processing of data was to remove noise from the collected data. The moving average filter (MAF) method is an effective technique by which to reduce random noise of time domain-encoded signals [23]. The effect of noise arising from mattress pressure can be minimized by calculating the MAF as follows:

1 $$ MAF  =  \frac{{{x}_{n} \,{ + }\, {x}_{{{n} - 1}} \,{ + }\,{x}_{n - 2} \,{ + } \cdots { + }\,{x}_{n - N + 1} }}{N} $$

where *x*_n_ is the *n*th time sensing data and N is the number of points (N = 10). After using the MAF, a thresholding filter was adopted to highlight essential sensing data. If the pressure/temperature value was greater than the defined threshold, the sensing value was assigned as 1; otherwise, the sensing value was assigned as 0. Data that exceeded the thresholds were termed “sensed points”.

### Middle point/axis determination using fuzzy c-means clustering

After pre-processing of the raw sensor data, the middle point and the middle axis needed to be calculated using a fuzzy c-means (FCM) clustering algorithm. FCM [24–26] is a widely-used method for soft image clustering, and was employed in this study to eliminate the effects of position and orientation differences of different subjects on sleeping posture determination. Given a horizontal/vertical axis and a dependent feature vector x, FCM aims to minimize the following objective function:

2 $$ A\, = \,\mathop \sum \limits_{\text{i = 1}}^{n} \mathop \sum \limits_{{{k = }1}}^{c} {u}_{ik}^{m} \left\| {x_{i} \, - \,{c}_{k}} \right\| ^{ 2} $$

where A is the objective function, *n* is the number of horizontal/vertical axes, *c* is the number of clusters, *u*_*ik*_ is the degree of membership of *x*_*i*_ in cluster *k*, *x*_*i*_ is the *i*th value of d-dimensional measured data, *c*_*k*_ is the d-dimension center of the *k*th cluster. The Euclidean distance between *x*_*i*_ and *c*_*k*_, $\left\| {x}_{i} - {c}_{k} \right\|$, can be calculated as:

3 $$ \left\|{x}_{i} \, - \,{c}_{k}\right\|  \,= \, \sqrt {\mathop \sum \limits_{i = 1}^{n} ( {x}_{iP} \, - \,{c}_{kP} )^{ 2} \,{ + }\, ( {x}_{iN} \, - \,{c}_{kN} )^{ 2} } $$

where *x*_*iP*_ and *c*_*kP*_ are positioning indexes on the horizontal/vertical axis of sensing points after histogram projection processing, and *x*_*iN*_ and *c*_*kN*_ are the numbers of sensed points. The degree membership *u*_*ik*_ can be calculated as:

4 $${u}_{ik} \, = \,\frac{ 1}{{\mathop \sum \nolimits_{{{l} = 1}}^{c} \left( {\frac{{\left\|{x}_{i} \, - \,{c}_{k}\right\| }}{{\left\|{x}_{i} \, - \,{c}_{l}\right\|}}} \right)^{{\frac{ 2}{{m} - 1}}} }} $$

The FCM clustering algorithm has five steps:

1. Set the number *c* of clusters and the stopping condition.
2. Calculate the cluster centroid.
3. For each sensor point, compute the membership value for each cluster.
4. Compute the objective function, shown in formula (2). If the value of *A* between consecutive iterations < ε, then stop. Otherwise, go to step 2.
5. Assign each positioning index to a cluster after defuzzification.

After building up the FCM clustering, it can be applied to find the middle point/axis. For determination of the middle point of the trunk, the proposed method consisted of five steps, as follows:

#### 1. Histogram projection

**Fig. 5:**
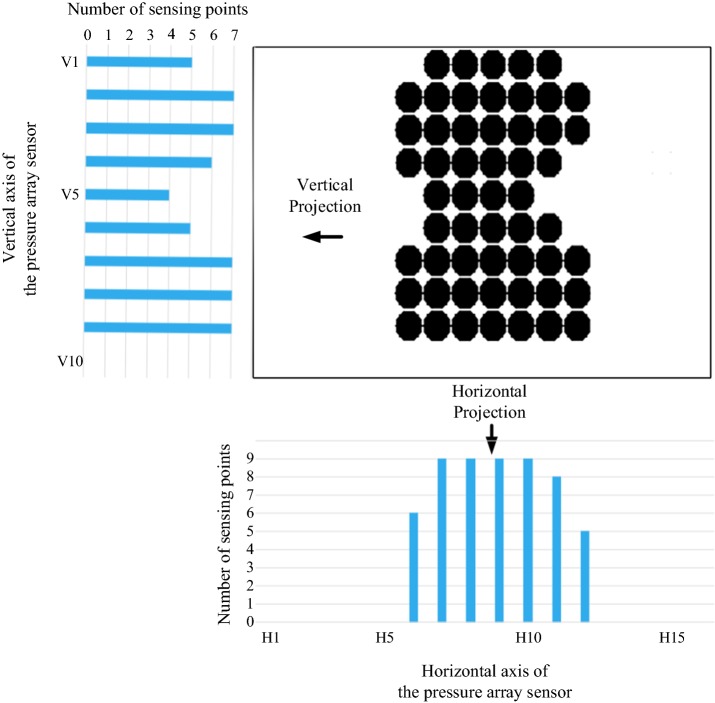
Histogram projections of vertical and horizontal axes

The data received by each pressure sensor is defined as a sensing point and the 2-dimension space of the bedsheet is defined as a coordinate. After pre-processing, the data of each sensing point are projected to horizontal and vertical directions, as shown in Fig. 5.

#### 2. Clustering

**Fig. 6:**
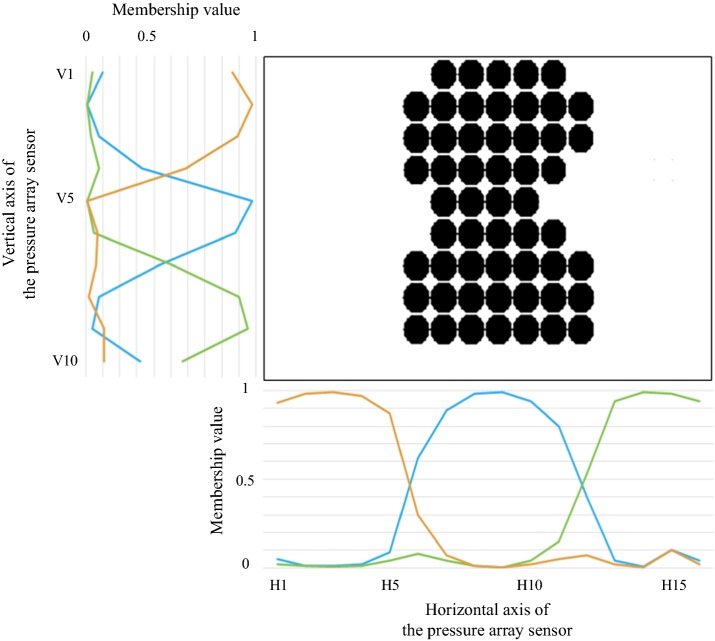
Middle point determination of the trunk using FCM

Using the FCM clustering algorithm, the Euclidean distance between *x*_*i*_ and *c*_*k*_ is calculated by considering axis positioning indexes and the number of projections, shown in formula (3). In other words, if the positioning indexes are closer to one another, they will be more likely to cluster into the same cluster; and if the number of projections on an axis positioning index is near to another, the axis positioning index will be more likely to cluster into the same cluster. The algorithm allows pre-definition of the number of clusters. In this study, the pressure sensors were clustered into three clusters in the horizontal and vertical directions, respectively (yellow, blue and green curves represent the three different clusters in Fig. 6).

#### 3. Finding the middle axis of the horizontal projection

Figure 6 shows the pressure sensors that detect the upper body. On the horizontal projection, the left-side cluster and the right-side cluster are both background clusters. The middle cluster is a foreground cluster mapped to the trunk, and the maximum membership value of the positioning index of the middle cluster is chosen as the middle axis. The same can be applied to the lower body to segment legs mapped into two sub-regions to find the middle axis of the leg map.

#### 4. Finding the middle axis of the vertical projection

On the vertical projection of Fig. 6, the middle cluster is mapped to the waist; the upper-side cluster is mapped to the chest, the lower-side cluster is mapped to the hips, and the maximum membership value of the positioning index of the middle cluster is chosen as the middle axis.

#### 5. Cross point of the two middle axes

Finally, by combining the two middle axes, the cross point can be determined. The cross point offers the key to segmenting the trunk map into four sub-regions.

### Feature extraction

**Fig. 7:**
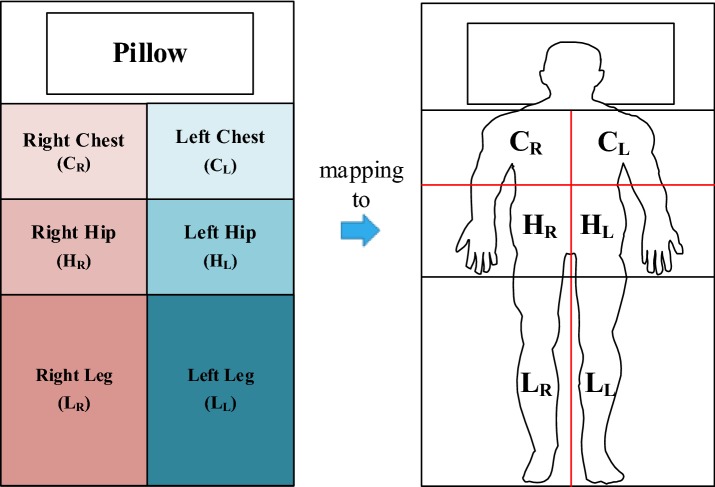
Body-part mapping

**Fig. 8:**
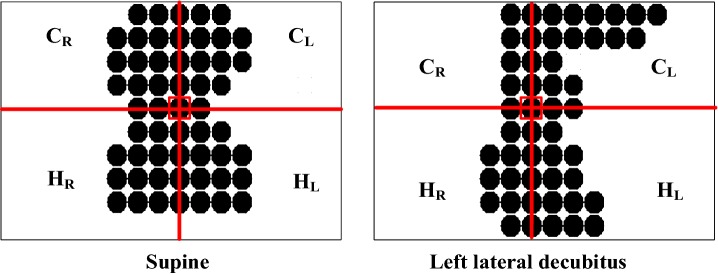
Pressure distributions of the supine and left lateral decubitus positions

**Fig. 9:**
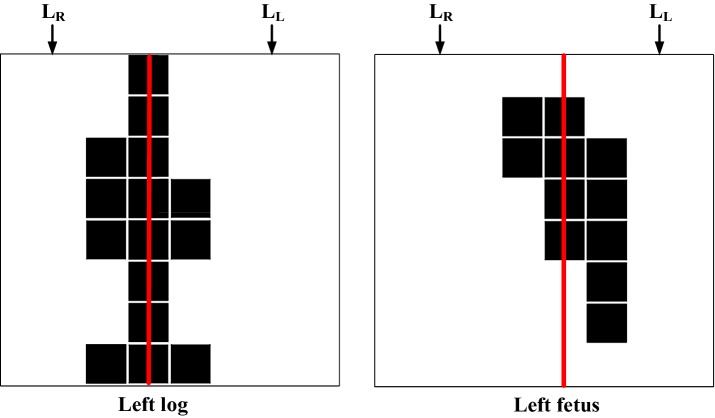
Temperature distributions of the left log and left fetus positions

The work in [18] proposed a feature extraction method for posture classification that is based on the geometry of the pressure images. The features are described as either spatial features or body-part features. Our feature extraction was based on the features of symmetry and balance. The middle point of the trunk and the middle axis of the legs were applied to segment the body-part map, as shown in Fig. 7. Four sub-regions were segmented by the middle point. In Figs. 8 and 9, the number of sensed points (*C*_*R*_, *H*_*R*_, *C*_*L*_, *H*_*L*_, *L*_*L*_, and *L*_*R*_) corresponds to each sub-region (right-chest, right-hip, left-chest, left-hip, left-leg, and right-leg), respectively. The symmetry feature of the body posture was extracted based on the sensed points of the four sub-regions. For example, when the subject’s posture was supine, the sensing pad observed more symmetrical features than for lateral postures. The four variables of the sub-regions of the body-part map were used to calculate the symmetry features, as shown below:

5 $$ {T}_{D - RL}  \, = \, ( {C}_{R}  \,+ \, {H}_{R} )\, - \,\left( {C}_{L} \, { + }\, {H}_{L}  \right) $$

6 $$ {T}_{D{ - CH}}  \,{ = }\, ( {C}_{R}  \,{ + }\, {C}_{L} )\, - \,\left( {{H}_{R}  \,{ + }\,{H}_{L} } \right) $$

7 $$ {L}_{D - RL}  \,{ = }\, \left| {{L}_{R} \, - \,{L}_{L} } \right| $$

8 $$ L_{MAC} \, = \,{\text{number of sensed points}} $$

where, *T*_*D*-*RL*_: Difference in number of sensed points between the right sub-regions $( {C}_{R}  \,{ + }\, {H}_{R} )$ and the left sub-regions $\left( {{C}_{L}  \,{ + }\, {H}_{L} } \right)$ in the trunk region. *T*_*D*-*CH*_: Difference in the number of sensed points between the chest sub-regions $( {C}_{R}  \,{ + }\, {C}_{L} )$ and the hip sub-regions $\left( {{H}_{R}  \,{ + }\, {H}_{L} } \right)$ in the trunk region. *L*_*D*-*RL*_: Absolute difference in the number of sensed points between the right sub-region $( {L}_{R} )$ and the left sub-region $\left( {{L}_{L} } \right)$ in the leg region. *L*_*MAC*_: Number of sensed points falling in the middle axis in the leg region.

Figure 8 demonstrates the pressure distributions of the supine and left lateral decubitus positions. The sensed points were distributed evenly in the supine position, while most of the sensed points fell in the left sub-regions (*C*_*L*_ and *H*_*L*_) in the left lateral decubitus position. The left–right symmetry measures were obtained by calculating the difference (*T*_*D*-*RL*_) between the right sensed point sum (*C*_*R*_+ *H*_*R*_) and the left sensed point sum (*C*_*L*_+ *H*_*L*_), as shown in Eq. (5). Similarly, the chest–hip symmetry measures were obtained by calculating the difference (*T*_*D*-*CH*_) between the chest sensed point sum (*C*_*R*_+ *C*_*L*_) and the hip sensed point sum (*H*_*R*_+ *H*_*L*_), as per Eq. (6).

Figure 9 demonstrates the thermal image distribution of the left log and left fetus positions. As indicated in the figure, the thermal image of a straight leg is represented by a straight line and looks symmetrical, while a curled-up leg appears asymmetrical. The left–right symmetry features were obtained by calculating the absolute value of the difference (*L*_*D*-*RL*_) between the number of sensed points in the right-leg region (*L*_*R*_) and the number of sensed points in the left-leg region (*L*_*L*_), as per Eq. (7). Equation (8) indicates the number of sensed points located in the middle axis of the legs.

### Sleeping posture classification using a decision tree

**Fig. 10:**
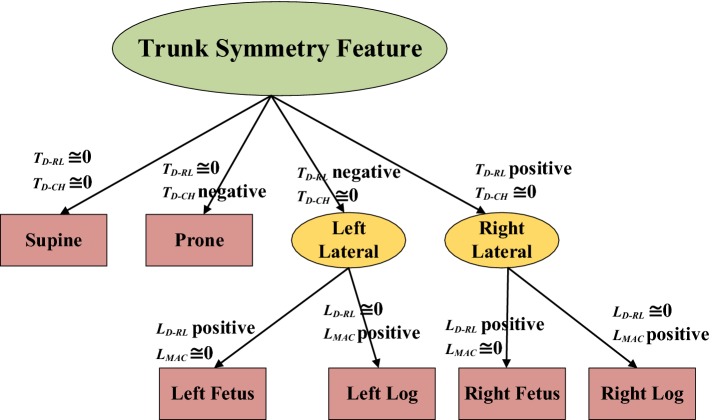
Sleeping posture classification using a decision tree

The decision tree method is one of the most common predictive modeling approaches used in statistics, data-mining and machine learning. We used a distance-weighted *k*-nearest neighbor algorithm (*k*-NN) [27] to classify the sleeping postures of a subject. In our decision tree structure, as shown in Fig. 10, leaves represented sleeping postures and branches represented conjunctions of features that led to those sleeping postures. Two feature spaces were applied: the trunk symmetry feature and the leg symmetry feature. In each feature space, we employed the *k*-NN classification technique to recognize the sleeping posture.

### Feature spaces of sleeping postures

**Fig. 11:**
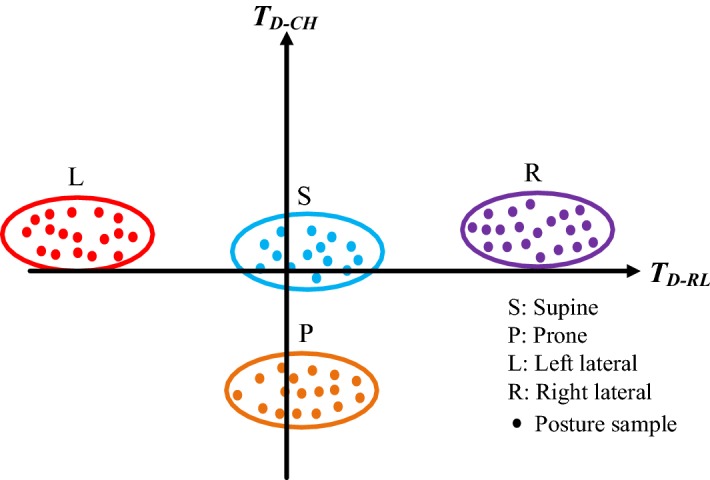
Feature space of trunk symmetry

**Fig. 12:**
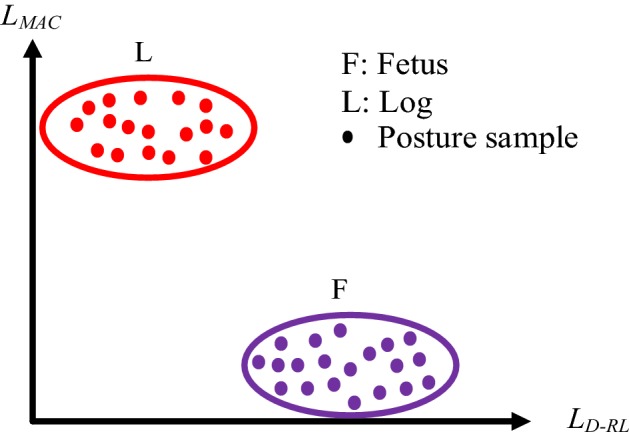
Feature space of leg symmetry

This study applied two kinds of feature space to recognize sleeping postures: the trunk symmetry feature space (Fig. 11) and the leg symmetry feature space (Fig. 12). Four major upper-body postures, consisting of supine, prone, left lateral and right lateral positions, were described by the trunk symmetry feature space. As described in previous sections, *T*_*D*-*RL*_ and *T*_*D*-*CH*_ were used to compose the trunk symmetry feature space. When the posture was supine, both *T*_*D*-*RL*_ and *T*_*D*-*CH*_ were close to zero (close to the origin of the space). When the posture was left lateral, *T*_*D*-*RL*_ was negative and *T*_*D*-*CH*_ close to zero. When the posture was right lateral, *T*_*D*-*RL*_ was positive and *T*_*D*-*CH*_ close to zero. When the posture was prone, *T*_*D*-*RL*_ was close to zero and *T*_*D*-*CH*_ was negative (close to the bottom of the space). The two major lower-body postures, the fetus and the log positions, are described by the leg symmetry feature space. As described above, *L*_*D*-*RL*_ and *L*_*MAC*_ were used to compose the leg symmetry feature space. When the posture was of the fetus position, *L*_*D*-*RL*_ was large and *L*_*MAC*_ was small (close to the right-hand bottom of the space). When the posture was of the log position, *L*_*D*-*RL*_ was small and *L*_*MAC*_ large (close to the left-hand upper limit of the space).

### Measurement of the sensing gap for sensor deployment on the bedsheet

**Fig. 13:**
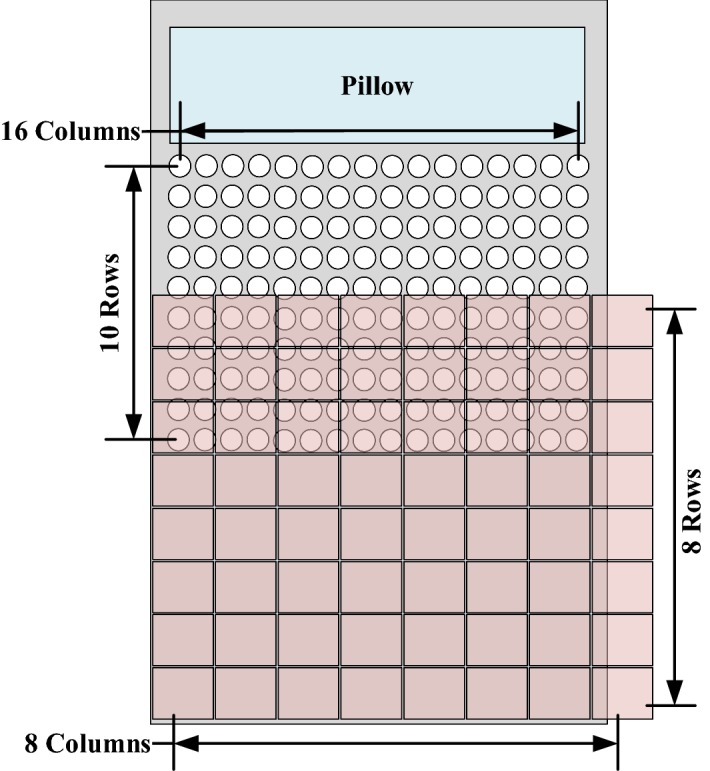
Experimental setup

**Fig. 14:**
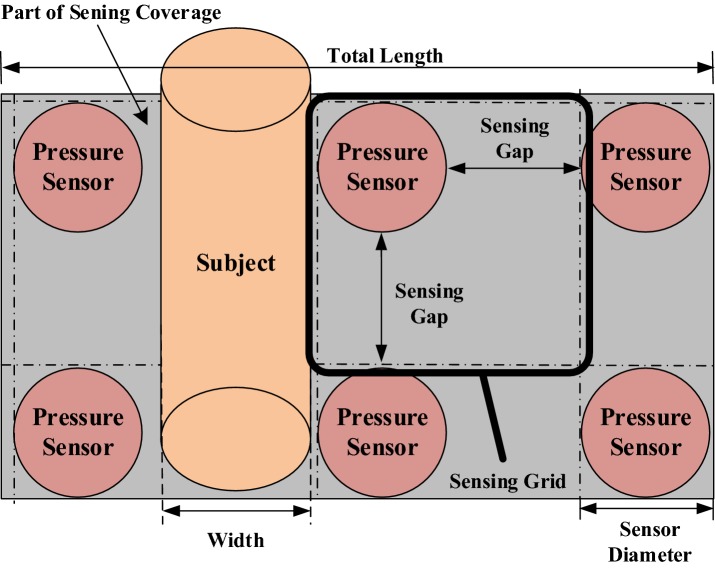
Sensing gap in pressure sensor arrays

**Fig. 15:**
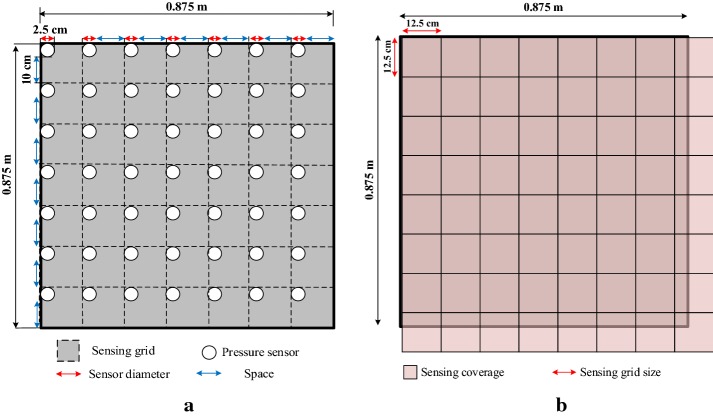
Deployment comparison: **a** pressure sensors; **b** infrared array sensor

Deploying fewer sensors in a fixed sensing coverage will lead to sensing gaps between sensors. We deployed a 16 × 10 pressure sensor array for the upper body to avoid creation of a sensing gap, as shown in Fig. 13. Sensing gaps occur when sensors are not adjacent to each other in sensor deployment. In such cases, there exist areas between sensors in which the system does not work when a subject’s body part is smaller than the area and falls within the area. In sensing coverage in which the size of the sensors is fixed, deployment of fewer sensors will create larger sensing gaps. Figure 14 shows that many sensing gaps exist between sensors. An area that contains a sensor and a gap is defined as a sensing grid. When a subject sleeps on the bedsheet, the major pressure distribution is contributed by the chest and hips; in other words, pressure sensors have a low utilization in the leg area, which is a waste of resources. To address the cost issue, previous studies usually deployed fewer sensors. A typical example is shown in Fig. 15a, which depicts a pressure sensor array deployed on a bedsheet. However, fewer sensors in the sensing area enlarge the gap between sensors. To resolve this problem, we adopted a Panasonic AMG8852 Grid-EYE [21] infrared array sensor for leg-posture recognition. The Grid-EYE system is an infrared thermal imaging sensor containing an 8 × 8 matrix of pixels deployed at a height of 0.96 m, which covers a sensing area of 1 m × 1 m.

Figure 15 presents a comparison of a typical pressure sensor deployment (Fig. 15a) and an infrared array sensor deployment (Fig. 15b). The sensor deployment area is crisscrossed with dotted lines that form sensing grids. To simplify the calculation process, the pressure sensors shown in Fig. 15a were attached to the upper and left dotted lines of each sensing grid, while in practice the force applied to the pressure sensors diffuses radially outward owing to the effect of the mattress covering the sensors.

### Spatial sensing resolution

The sensing gaps created during pressure sensor and infrared array sensor deployment were compared. In order to make a fair comparison, a similar spatial resolution was considered for both the FSR and the Grid-EYE sensors. In [19], spatial sensing resolution was proposed as a tool for comparison of three different pressure sensor deployments. For the pressure sensor deployment shown in Fig. 15a, the spatial sensing resolution is shown in Eq. (9):

9 $$ \begin{aligned} {\text{Spatial sensing resolution}} & = {\text{number of sensing points}}/{\text{area of sensing coverage}} \\ & = \, {{\left( { 7 { } \times { 7}} \right)} \mathord{\left/ {\vphantom {{\left( { 7 { } \times { 7}} \right)} {\left( {0. 8 7 5 { } \times \, 0. 8 7 5} \right)}}} \right. \kern-0pt} {\left( {0. 8 7 5 { } \times \, 0. 8 7 5} \right)}} \\ & = { 64 }\left( {{{sensing \, points} \mathord{\left/ {\vphantom {{sensing \, points} {{\text{m}}^{ 2} }}} \right. \kern-0pt} {{\text{m}}^{ 2} }}} \right) \\ \end{aligned} $$

However, the spatial sensing resolution for the deployment of an infrared array sensor of the same coverage (Fig. 15b) is given by:

10 $$ \begin{aligned} {\text{Spatial sensing resolution}} & = {\text{ number of sensing pixels}}/{\text{area of sensing coverage}} \\ & = \, {{\left( { 8\times 8} \right)} \mathord{\left/ {\vphantom {{\left( { 8\times 8} \right)} {\left( { 1\times 1} \right)}}} \right. \kern-0pt} {\left( { 1\times 1} \right)}} \, = { 64 }\left( {{{sensing \, pixels} \mathord{\left/ {\vphantom {{sensing \, pixels} {{\text{m}}^{ 2} }}} \right. \kern-0pt} {{\text{m}}^{ 2} }}} \right) \\ \end{aligned} $$

The sensing gap of the pressure sensor array deployment was calculated for the array shown in Fig. 15a as 0.1 m. Sensing grids of every two adjacent or diagonal pixels of the Grid-EYE system adjoin each other, sharing a common border or endpoint. The sensing coverage of each pixel will change if the distance between the Grid-EYE array and the subject varies, meaning that there exists no obvious sensing gap in the Grid-EYE pixel array.

### Signal detection threshold

The signal detection threshold is the largest body part of a subject that is undetectable by the deployed sensor(s) in a sensing system. In this study, the signal detection threshold was applied to evaluate the capability of forming a normal-size human leg contour on the sensing bedsheet. In common usage, a *threshold* is the minimal amount of a subject’s feature necessary to be detectable by the sensory receptor or system [28]. When value of the feature (e.g., brightness, weight, temperature) reaches or exceeds a certain threshold, the system will detect the existence of the feature. In our evaluation of a sensing bedsheet system, the maximal undetectable size of the subject within the sensors was considered “the worst case” in the sensing grid. The *signal detection threshold* in a sensing grid is described by Eq. (11):

11 $$ {Signal\, detection \,threshold = {\text{max}}(A}_{u} ) $$

where $${A}_{u} $$ is an undetectable area of a subject’s body part.

The quality of sensor deployment can be examined using the value of the signal detection threshold. When the undetectable area reaches the maximal value, which is its signal detection threshold, the ratio of the size of the maximal value to the sensing grid reflects the quality of sensor deployment. In the equation, $A_{u}$ is the undetectable area of the subject’s body part on the sensing bedsheet. The area will keep increasing until it hits a sensor and is detected by that sensor. Therefore, the largest size of body part that is not detected by any of the sensors, which is the ${\text{max}}(A_{u} )$, is the signal detection threshold. The signal detection threshold is better when the value is smaller.

### Pressure sensor array deployment

**Fig. 16:**
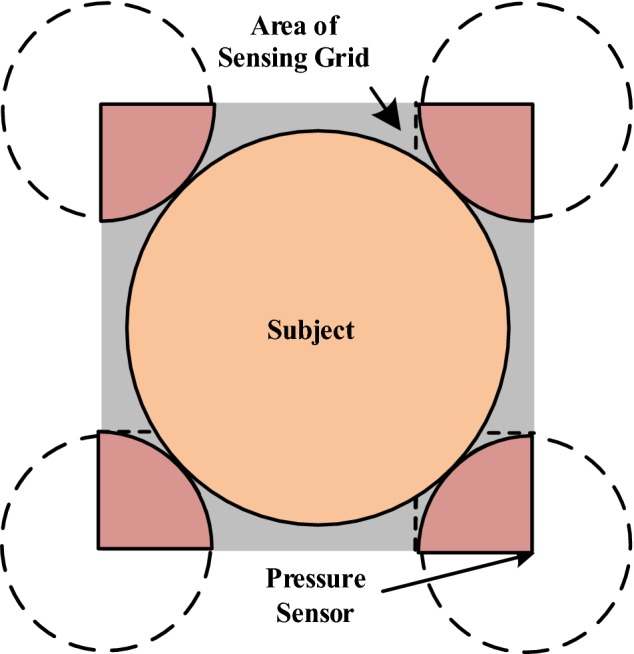
Sensing grid of pressure sensor array deployment for signal detection threshold calculation

In a pressure sensor array deployment, the sensing grid is the minimum value of a quadrilateral area that includes the center of every four sensors of an in-bed pressure sensor deployment. “The worst case” position means that a body part of the subject falling within the area of four pressure sensors has the largest undetectable feature area. The subject touches the four sensors but does not overlap any of them, rendering it undetectable. A sensing unit containing four quarter circles and the largest-but-not-overlapping subject’s body part is shown in Fig. 16; this figure is another demonstration of the ${\text{max(}}A_{u} )$, in which the sensing gap and the subject’s body part are placed in the middle of the sensing grid. According to the experimental results, the signal detection threshold (i.e., the maximum undetectable area of a body part of the subject) was approximately $1 8 0. 8 3   {\text{cm}}^{ 2}$.

### Infrared array sensor deployment

In an infrared array sensor deployment, the signal detection threshold is the smallest detectable area of a body part of the subject within the sensing grid. Our Grid-EYE experiments showed that a shank was undetectable when it fell in one-fifth (20 cm^2^) of the sensing grid, but detectable in one-quarter (25 cm^2^) of the sensing grid. Similarly, when two fingers (18 cm^2^) fell in the Grid-EYE sensing grid, they were undetectable; however, three fingers (27 cm^2^) were detectable in the sensing grid. According to the experimental results, the ${\text{max(}}A_{u} )$ for the Grid-EYE system was approximately 25 cm^2^.

Table 1 presents a comparison of the pressure sensor array (FSR sensors) and infrared array sensor deployment in the leg area. The calculations showed that under the same spatial resolution, the signal detection threshold of the Grid-EYE system was much better than that of the pressure sensor array (FSR sensors). A single infrared sensor covers a larger sensing area than 49 FSR sensors. The pressure distribution of the legs is very small, and this decreases the accuracy of the FSR-based method. Hence, combining FSR sensors and infrared array sensors renders posture recognition effective and cost-efficient.

**Table 1:** Comparison of FSR sensor and infrared array sensor deployment

	Number of sensors	Sensing gap	$$ {\text{max(}}\varvec{A}_{\varvec{u}} ) $$	Cost
Pressure sensor array	49	0.1 m	$$ 1 8 0. 8 3 \,{\text{cm}}^{ 2} $$	$340
Proposed infrared array sensor	1	N/A	$$ 2 5 \, {\text{cm}}^{ 2} $$	$34

The quality of sensor deployment was better when the value of ${\text{max(}}A_{u} )$ was smaller

## Results and discussion

We performed experiments with different subjects based on their body weight: light (around 40 kg), medium (around 60 kg), and heavy (around 80 kg). The sleeping posture recognition accuracy of the proposed method was as shown in Table 2; the average sleeping posture recognition accuracy was 88.05%.

**Table 2:** Accuracy of the proposed method with different subject body weights

Body weight	Accuracy of proposed method (%)
Light (around 40 kg)	90
Medium (around 60 kg)	85.83
Heavy (around 80 kg)	88.33
Average	88.05

We compared the number of deployed sensors and the cost of the method with systems described in previous studies, as shown in Table 3. In [17, 18], 8192 FSR sensors were deployed, achieving an accuracy of 90.78% and 83%, respectively. The cost of 8192 FSR sensors was approximately 8850 USD. Our proposed scheme employs 160 FSR sensors for the upper body and one infrared array sensor for the lower body, the total cost of which was approximately 950 USD. We achieved an accuracy of 88.05% using very few pressure sensors. In the experiment, an infrared array sensor system was chosen instead of a pressure sensor system for detection of the position of the lower part of the body. When sensor deployment only is taken into consideration, the cost of an infrared array sensor system is about one-tenth that of a pressure sensor system. Additionally, in comparison to an infrared array sensor, pressure sensor systems have a much higher maintenance cost, as patients do not come into physical contact with infrared sensors. A hybrid deployment consisting of an infrared array sensor for the lower body effectively reduces the deployment cost.

**Table 3:** Comparison of sleeping posture recognition methods

	Cost	Number of sensors	Accuracy (%)
Xu et al. [17]	High	8192 pressure sensors	90.78
Liu et al. [16]	High	8192 pressure sensors	83
Proposed method	Low	160 pressure sensors and 1 infrared array sensor	88.05

The proposed sensor deployment was found to be much more cost-effective as compared with the number of pressure sensors deployed in [17] and [18]. The experimental results indicated that our proposed system achieved highly accurate posture recognition at a much lower cost.

## Conclusions

In this study, we designed and tested a sleeping posture recognition scheme consisting of a pressure sensor array and an infrared array sensor. We deployed 16 × 10 FSR sensors for the upper body and a Grid-EYE infrared array sensor for the lower body to create a bed sensing system. We applied the symmetry of feature space to distinguish six types of sleeping body posture. The proposed method overcame lying position variations by determining the middle point and middle axis of the body. The experimental results showed the accuracy of posture recognition to be 88.05%.

As we deployed FSR sensors only on the upper bedsheet, and employed a single infrared array sensor for the lower body, the novel scheme was cost-effective. Moreover, the proposed scheme avoided sensing gaps between sensors, which is a common issue but is usually ignored in low-cost pressure sensor systems. The Grid-EYE infrared array sensor deployment balanced the trade-off between recognition accuracy and sensor cost. In addition, the Grid-EYE sensor deployment negated privacy concerns.

## References

[1] Hsiao RS, Mi Z, Yang BR, Kau LJ, Bitew MA, Li TY (2015). Body posture recognition and turning recording system for the care of bed bound patients. Technol Health Care.

[2] Tangtrakulwanich B, Kapkird A (2012). Analyses of possible risk factors for subacromial impingement syndrome. World J Orthop..

[3] Lee JB, Park YH, Hong JH, Lee SH, Jung KH, Kim JH, Yi H, Shin C (2009). Determining optimal sleep position in patients with positional sleep-disordered breathing using response surface analysis. J Sleep Res.

[4] Cheyne JA (2002). Situational factors affecting sleep paralysis and associated hallucinations: position and timing effects. J Sleep Res.

[5] Johnson DA, Orr WC, Crawley JA, Traxler B, McCullough J, Brown KA, Roth T (2005). Effect of esomeprazole on nighttime heartburn and sleep quality in patients with GERD: a randomized, placebo-controlled trial. Am J Gastroenterol.

[6] van Herwaarden MA, Katzka DA, Smout AJPM, Samsom M, Gideon M, Castell DO (2000). Effect of different recumbent positions on postprandial gastroesophageal reflux in normal subjects. Am J Gastroenterol.

[7] Hao T, Xing G, Zhou G, iSleep: Unobtrusive Sleep Quality Monitoring using Smartphones. In: Proceedings of the 8th ACM conference on embedded networked sensor systems (SenSys). ACM; 2013, p. 1–14.

[8] De KJ, Gagnon P, Lallier S (1983). Sleep positions in the young adult and their relationship with the subjective quality of sleep. Sleep.

[9] Lee H, Xie L, Yu M, Kang H, Feng T, Deane R, Logan J, Nedergaard M, Benveniste H (2015). The effect of body posture on brain glymphatic transport. J Neurosci.

[10] Hsia CC, Hung YW, Chiu YH, Kang CH. Bayesian classification for bed posture detection based on kurtosis and skewness estimation. In: 2008 10th IEEE international conference on e-health networking, applications and services (HealthCom). IEEE; 2008. p. 165–8.

[11] Chang KM, Liu SH (2011). Wireless portable electrocardiogram and a tri-axis accelerometer implementation and application on sleep activity monitoring. Telemed J E Health..

[12] Hoque E, Dickerson RF, Stankovic JA. Monitoring body positions and movements during sleep using wisps. In: Wire-less health 2010 (WH ‘10). ACM; 2010, p. 44–53.

[13] Yu M, Rhuma A, Naqvi SM, Wang L, Chambers J (2012). A posture recognition-based fall detection system for monitoring an elderly person in a smart home environment. IEEE Trans Inf Technol Biomed.

[14] Bhatia S, Sigal L, Isard M, Black MJ. 3D human limb detection using space carving and multi-view eigen models. In: 2004 IEEE conference on computer vision and pattern recognition workshop (CVPRW’04). IEEE; 2004.

[15] Chen CC, Hsieh JW, Hsu YT, Huang CY. Segmentation of human body parts using deformable triangulation. In: 18th international conference on pattern recognition (ICPR’06). 2006. p. 355–8.

[16] Liu JJ, Xu W, Huang MC, Alshurafa N, Sarrafzadeh M, Raut N, Yadegar B. A dense pressure sensitive bedsheet design for unobtrusive sleep posture monitoring. In: 2013 IEEE international conference on Inpervasive computing and communications (PerCom). IEEE; 2013. p. 207–15.

[17] Xu X, Lin F, Wang A, Song C, Hu Y, Xu W. On-bed sleep posture recognition based on body-earth mover’s distance. In: Biomedical circuits and systems conference 2015. (BioCAS). IEEE; 2015. p. 1–4. 10.1109/TBCAS.2016.2543686 27483475

[18] Liu JJ, Xu W, Huang MC, Alshurafaa N, Sarrafzadeha M, Rautc N, Yadegarc B (2014). Sleep posture analysis using a dense pressure sensitive bedsheet. Pervasive Mob Comput..

[19] Wai A, Foo S, Huang W, Biswas J, Hsia CC, Liou K, Yap P (2010). Lying posture classification for pressure ulcer prevention. J Healthc Eng..

[20] General Purpose 2.5 cm-diameter ultra-thin flexible pressure sensor. Uneo Sensor. 2015. http://www.uneotech.com/uneo/images/upload/GD25-100N%20ENG.pdf. Accessed 13 Nov 2016.

[21] Infrared Array Sensor Grid-EYE. Panasonic. 2014. http://industrial.panasonic.com/ww/products/sensors/built-in-sensors/grid-eye, Accessed 13 Nov 2016.

[22] Idzikowski C (2003). Beating insomnia: how to get a good night’s sleep.

[23] Smith SW (2006). The scientist and engineer’s guide to digital signal processing.

[24] James CB (2013). Pattern recognition with fuzzy objective function algorithms.

[25] Hung YW, Chiu YH, Jou YC, Chen WH, Cheng KS (2015). Bed posture classification based on artificial neural network using fuzzy c-means and latent semantic analysis. J Chin Inst Eng.

[26] Kiran M, Lai WK, Kyaw K, Ali H. Clustering techniques for human posture recognition: K-means, FCM and SOM. In: SSIP’09/MIV’09 9th WSEAS international conference on signal, speech and image processing, and 9th WSEAS international conference on multimedia, internet and video technologies. 2009. p. 63–7.

[27] Hu F, Hao Q (2012). Intelligent sensor networks: the integration of sensor networks, signal processing and machine learning.

[28] Galantner R, Hess E, Brown EH, Brown R, Galanter E, Hess EH, Mandler G (1962). Contemporary psychophysics. New directions in psychology.

